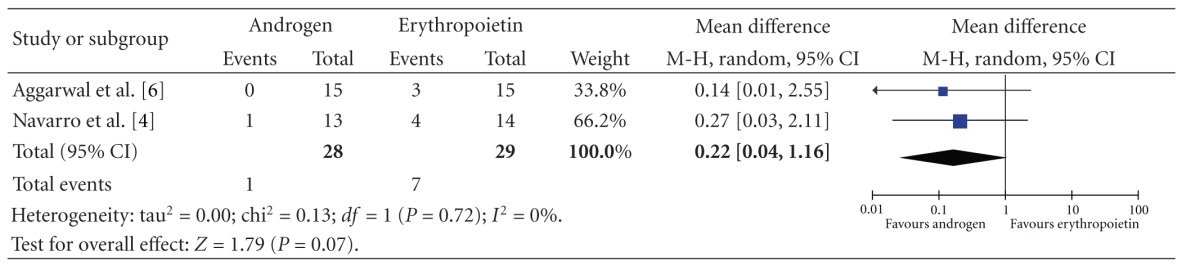# Meta-Analysis of Randomized Controlled Trials on Androgens versus Erythropoietin for Anaemia of Chronic Kidney Disease: Implications for Developing Countries

**DOI:** 10.1155/2012/580437

**Published:** 2012-10-16

**Authors:** B. Adamu, S. M. Ma'aji, P. J. Erwin, I. M. Tleyjeh

**Affiliations:** ^1^Nephrology Unit, Department of Medicine, Bayero University, Kano, PMB 3452, Nigeria; ^2^Usmanu Danfodiyo University, Sokoto, PMB 2346, Nigeria; ^3^Mayo Clinic College of Medicine, Rochester, MN 55905, USA; ^4^King Fahad Medical City, Riyadh 11525, Saudi Arabia

## Abstract

Androgens which are relatively cheap were used in the treatment of anaemia in dialysis patients before the advent of Erythropoietin (EPO). However, there are concerns about their efficacy and side effects. *Aims*. To examine the efficacy and harms of androgens for the treatment of anaemia of chronic kidney disease (CKD) compared to EPO. *Settings and Design*. A systematic review and meta-analysis using an a priori protocol. *Methods and Materials*. We searched several databases for randomized controlled trials using the key terms anaemia, chronic kidney disease, and androgens, without language restrictions. We also searched reference lists of relevant articles. *Statistical Analysis Used*. Data was analyzed using Review manger 5 software. We summarized treatment effects as relative risks and mean differences, with 95% confidence intervals using a random-effect model. We tested for heterogeneity with Chi^2^ and the *I* ^2^ statistics. *Results*. We identified four eligible trials involving 114 participants, majority (83.33%) of whom were males, mostly over 50 years of age. The pooled difference in mean haemoglobin between the nandrolone and EPO arms at the end of the trials was −0.11 (CI −0.80 to 0.58) which is not statistically significant. *Conclusions*. This meta-analysis revealed no difference between nandrolone and EPO for the treatment of anaemia of CKD in men over 50 years. Therefore, nandrolone can be used for the treatment of anaemia of CKD in this category of patients, in resource-limited countries. However, further studies are needed to determine the long-term safety of nandrolone in men over 50 years old, as well as its effectiveness and safety in females in general, and males less than 50 years of age.

## 1. Introduction

Erythropoietin (EPO) and other erythropoiesis stimulating agents (ESAs) are the main stay for the treatment of anaemia of chronic kidney disease (CKD). The main limitation of EPO use in developing countries is cost, making it unavailable to most patients [1, 2]. Androgens which are relatively cheap were used in the treatment of anaemia in dialysis patients before the advent of EPO [3]. However, there are concerns about their efficacy and side effects. The aim of this systematic review and meta-analysis was to examine the efficacy and harms of androgens for the treatment of anaemia of CKD compared to EPO from published randomized controlled trials.

## 2. Subjects and Methods 

The study was carried out using an a priori protocol. We searched several databases (MEDLINE, EMBASE, The Cochrane library, LILACS, AJOL, and CINAHL ) for randomized controlled trials using the key terms anaemia, chronic kidney disease, and androgens, without language restrictions. We also searched a reference list of relevant articles. Inclusion criteria are randomized or quasi-randomized controlled trials that directly compared any androgen with EPO for the treatment of anaemia of CKD. Exclusion criteria were study designs other than RCTs or quasi-RCTs and studies where androgens were used as adjuvants for the treatment of anaemia. In trials where there is more than two arms of treatment (e.g., androgen versus EPO versus androgen plus EPO), we extracted data for only androgens and the EPO arms.

The primary outcome of interest in this paper is the mean haemoglobin at the end of the trials. Secondary outcomes include potential side effects in the two treatment arms as reported by the authors of the primary studies. Other secondary outcomes include nutritional parameters such as weight gain and change in serum proteins.

Two authors independently selected relevant trials from the search results. Disagreements were resolved by consensus. Data were extracted using data extraction forms and analyzed using computer software, Review manager 5.1 (Cochrane collaboration). We assessed methodological qualities of the study using the risk of bias table. We summarized treatment effects as relative risks for dichotomous outcomes and mean differences for continuous outcomes with 95% confidence intervals using a random-effect model. We tested for heterogeneity with Chi^2^. We used the *I* ^2^ statistics to quantify between-study inconsistency.

## 3. Results

**Figure 1:**
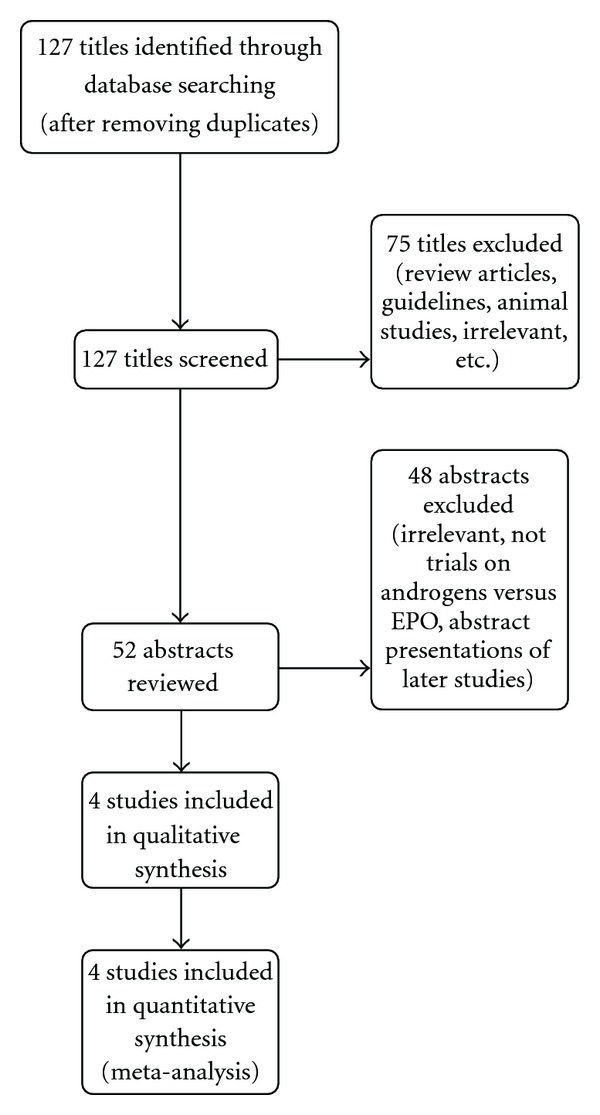


A total of 127 titles were obtained from searches of data bases out of which we identified four eligible trials (Figure 1).

**Figure 2:**
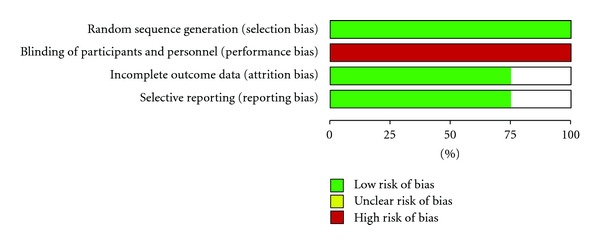
Representing Authors' methodological assessment of risk of bias across the studies.

The four studies [4–7] included in the meta-analysis had a total of 114 participants as summarized in Table 1. Data was extracted from full text of three studies while one study was only available in an abstract form. Overall, majority of the participants (83.33%) were males, mostly over 50 years of age. All the trials had small sample sizes (24–30 patients) with a follow-up period of three to six months. Methodological qualities of the studies were assessed using the risk of bias table in Review manager 5.1 (Cochrane collaboration). The qualities are presented in Figure 2.

There is a significant risk of bias since they are all open labelled studies with potential risk of performance bias.

Eighty-three % of the patients in the studies (95 out of 114) were males (most of them over 50 years of age) while two studies included few females (19 out of 114) and patients younger than 50 years. Nandrolone was the only androgen used in all the trials, at the dose of 100–200 mg/week intramuscularly. 

The pooled difference in mean haemoglobin between the androgen and EPO arms at the end of the trials was −0.11 (CI −0.80 to 0.58) which was not statistically significant (Table 2).

Although there was significant heterogeneity for the outcome of haemoglobin across studies (*I* ^2^ of 82%), all individual effect estimates did not favour EPO over androgens (neither clinically meaningful nor statistically significant estimates).

Three trials reported increase in total serum protein which favours androgens with a mean difference of 0.54 (CI 0.4 to 0.68) which is statistically significant (Table 3).

There is a trend towards less increase in blood pressure requiring adjustment of blood pressure medications in the androgen arm with a relative risk of 0.17 ( CI 0.03–1.08), *P* = 0.06, although the difference is not statistically significant (Table 4).

No liver function abnormalities were encountered in both arms of the treatments in all the trials. Only one trial each presented some data on dyslipidemia and virilization, so meta-analysis could not be carried out for these outcomes. There was no significant heterogeneity for these outcomes. Three trials reported iron studies conducted in the participants while one study did not.

## 4. Discussion

In this systematic review and meta-analysis of randomized controlled trials, we found no clinically important or statistically significant difference between androgens (nandrolone) and EPO for the treatment of anaemia of CKD especially in men over 50 years. There is a statistically significant increase in serum proteins in the androgen arm of treatment. Moreover, nandrolone was associated with a trend to less hypertension events requiring changes in antihypertensive medications. Despite the limited available data, our findings have major implications for the care patients with CKD living in countries with limited resources.

 Anaemia is an important complication of CKD which can occur even at early stages of the disease [8] and is important both from the point of view of morbidity and mortality [9]. Erythropoietin and other related erythropoiesis stimulating agents (ESAs) are currently the main stay of the treatment of anaemia of CKD. However, recently there are concerns that the use of ESAs in the treatment of anaemia in CKD needs to be reevaluated [10].

Cost is a major limitation for the routine use of ESAs in developing countries. In a study from Tunisia, only 10.8% of patients on hemodialysis were on EPO, while 38% required regular transfusions [2].

In developing countries where patients cannot afford EPO, anaemia is treated mainly with recurrent blood transfusions with attendant risks of complications such as transfusion transmissible infections, especially in the current pandemic of human immune deficiency virus infection.

Prior to the advent of EPO in the 1980s and subsequently other ESAs, androgens such as nandrolone were used in the treatment of anaemia of CKD. Androgens are thought to correct anaemia of CKD by enhancing the conversion of the pluripotent stem cell to erythroid colony forming and burst forming units. In addition, 5-*α* metabolite of androgens stimulates erythropoiesis by enhancing erythropoietin production by the kidney, while the 5-*β* metabolite stimulates the bone marrow directly [11]. However, there are concerns about their efficacy and potential side effects such as hepatotoxicity, dyslipidemia, virilization, priapism, and hyperglycaemia [12]. In the current era of evidence-based medicine, it is important to study systematically the efficacy and potential side effects of androgens for the treatment of anaemia of CKD before sanctioning or discouraging their use. This is particularly important for many developing countries where patients cannot afford to buy ESAs. Androgens are much cheaper than ESAs being five-times cheaper than EPO treatment in the study by Aggarwal et al. [6].

Our meta-analysis has some limitations. One limitation is the fact that the findings are not generalizable to all patient populations with CKD. Two of the four studies included in this meta-analysis had only male patients over the age of 50, likely because response to androgens has previously been shown to be age related [13] and because of the risk of virilization in females. Androgens may be more effective in older males because of testosterone deficiency in these patients. Therefore, the findings of this study may not be applicable to women or men under 50 years of age.

Although there are no significant differences in side effects reported in the studies in this meta-analysis, this has to be interpreted with caution. The number of patients in the trials included in this meta-analysis is too small to exclude safety concerns. The trials had follow-up periods of three to six months and therefore may not have been sufficient to detect some of the known side effects of androgens which may take longer to develop.

Another potential limitation of this meta-analysis is the high degree of heterogeneity for the outcome of haemoglobin at the end of the trials. However, all individual effect estimates did not favour EPO over androgens (neither clinically meaningful nor statistically significant estimates). Since we found no difference between the 2 groups, this heterogeneity is not meaningful. The small number of studies found in this meta-analysis is also a limitation as it precluded exploring for publication bias. The Cochrane handbook of systematic reviews recommends that as a rule of thumb: the funnel plot for detection of publication bias should not be used unless there are at least 10 studies [14]. Other formal tests for publication bias require even a larger number of studies (roughly 30) to reliably detect publication bias [15]. However, although the number of eligible studies found in this meta-analysis is small, other nonradomized trials support the findings of our study. Teruel et al. compared nandrolone in hemodialyzed males aged over 50 years with hemodialyzed males and females aged less than 50 years and found no difference in improvement of anemia between the two groups [16]. In a recent study in predialysis patients, Paul et al. concluded that nandrolone, though not equally effective, may be considered as a valid alternative therapy for the treatment of anemia of predialysis diabetic chronic kidney disease [17].

Our meta-analysis found a statistically significant advantage of androgens over EPO in terms of improvement in serum proteins which could be a marker of better nutrition. This is an additional advantage of nandrolone over EPO, which has also been supported by randomized controlled trials that address this and related outcomes [18, 19].

In addition to their role in the primary treatment of anaemia of CKD, androgens such as nandrolone have been used as adjuvants to the treatment of anaemia of CKD and for the treatment of erythropoietin-resistant anaemia [20, 21].

An ongoing systematic review in the Cochrane collaboration will hopefully critically appraise all these aspects of androgen use in anaemia of CKD [22].

We conclude that the results of this meta-analysis revealed no difference between the androgen nandrolone and EPO for the treatment of anaemia of CKD in men over 50 years. Therefore, androgens can be used for the treatment of anaemia of CKD in this category of patients, in resource-limited countries. However, further studies are needed to determine their long-term safety in men over 50 years old, as well as their effectiveness and safety in females in general, and males less than 50 years of age.

## Figures and Tables

**Table 1:** Characteristics of included studies.

Author	Country	Setting	Number of patients			Mean age + SD			Dose of nandrolone	Duration	Iron studies
			Males	Females	Total	Nandrolone	EPO	Total			
Navarro et al. [4]	Spain	CAPD	27	0	27	62 ± 7.00	60 ± 8.00	61 ± 7.00	200 mg/week	3 months	NR
Gascon et al. [5]	Spain	Hemodialysis	26	7	33	70 ± 4.00	72 ± 4.00	NR	200 mg/week	6 months	NS
Aggarwal et al. [6]	India	Predialysis	30	0	30	46 ± 12.61	49.90 ± 13.80	NR	100 mg/week	3 months	NS
Singh et al. [7]	India	Predialysis	12	12	24	NR	NR	NR	200 mg/week	6 months	NS

Key: CAPD: continuous ambulatory peritoneal dialysis, EPO: erythropoietin, NR: not reported, Iron studies: differences in iron studies at baseline between nandrolone group and EPO group, and NS: not significant.

**Table 2:** Mean haemoglobin at the end of the trials.

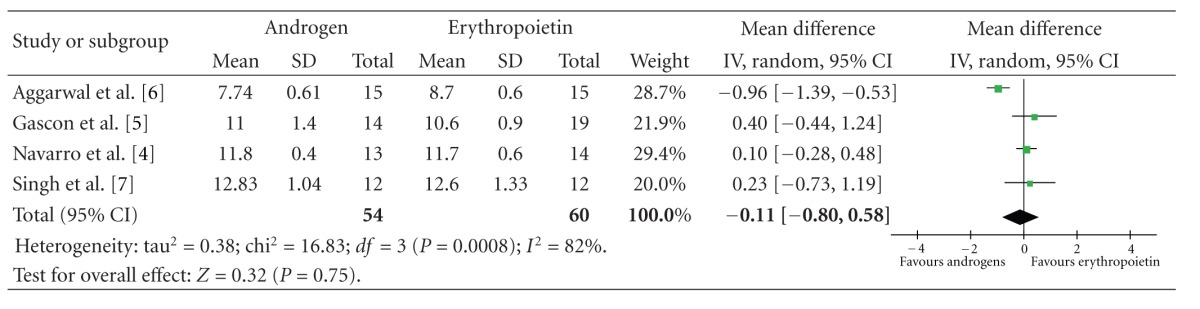

**Table 3:** Mean serum proteins at the end of the studies.

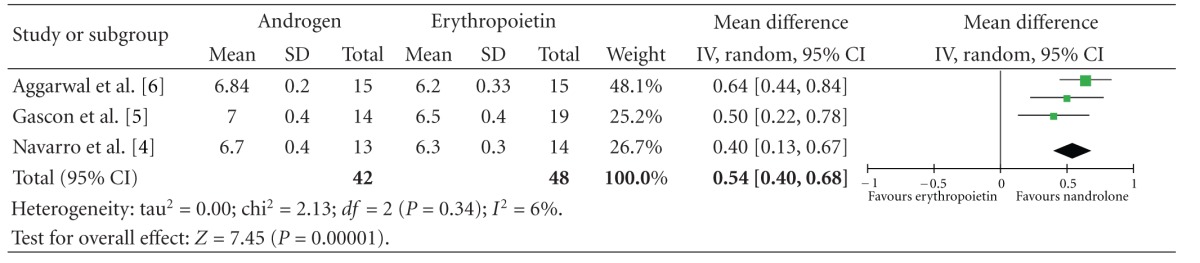

**Table 4:** Change in blood pressure requiring adjustment of antihypertensives.